# Quercetin as a Precursor for the Synthesis of Novel Nanoscale Cu (II) Complex as a Catalyst for Alcohol Oxidation with High Antibacterial Activity

**DOI:** 10.1155/2021/8818452

**Published:** 2021-03-03

**Authors:** Zahra Moodi, Ghodsieh Bagherzade, Janny. Peters

**Affiliations:** ^1^Department of Chemistry, University of Birjand, Birjand 97175-615, Iran; ^2^Department of Plant Systems Physiology, Radboud University Nijmegen, Nijmegen 6525, AJ, Netherlands

## Abstract

Quercetin (3,3′,4′,5,7-pentahydroxyflavone) is one of the dietary flavonoids, distributed in medicinal plants, vegetables, and fruits. Quercetin has the ability to bind with several metal ions to increase its biological activities. In the last two decades, quercetin has attracted considerable attention due to the biological and pharmaceutical activities such as antioxidant, antibacterial, and anticancer. In the present study, quercetin and ethanolamine were used for the synthesis Schiff base complex, which was characterized by IR, ^1^H NMR, and ^13^C NMR spectroscopy. The Schiff base has been employed as a ligand for the synthesis of novel nanoscale Cu (II) complex. The product was characterized by FT-IR spectroscopy, FESEM, and XRD. Significantly, the product showed remarkable catalytic activity towards the oxidation of primary and secondary alcohols. The antibacterial activity of the final product was assessed against *Staphylococcus aureus* (Gram‐positive) and *Escherichia coli* (Gram‐negative) bacteria using an inhibition zone test. The synthesized nanoscale Cu (II) complex exhibited a strong antibacterial activity against both Gram-positive and Gram-negative bacteria.

## 1. Introduction

**Figure 1:**
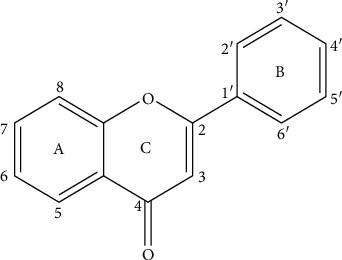
Basic skeleton of flavonoids.

Flavonoids are the largest group of phenolic compounds that have different biological and medicinal properties, such as antioxidant, [1] antibacterial, [2] antidiabetic, [3] anticancer, [4–6] antiatherosclerosis [7], and neuroprotective effects [8]. Flavonoids consist of two benzene rings joined by a 3-carbon bridge (C6-C3-C6) (Figure 1) [9]. Flavonoids can be divided into several different classes, such as flavones (e.g., flavone, luteolin, and apigenin), flavonols (e.g., quercetin, kaempferol, fisetin, and myricetin), and flavanones (e.g., flavanone, naringenin, and hesperetin). These classes are different in the oxidation and pattern of substitution of the C ring, while in each class, they differ in the pattern of substitution of the A and B rings [10].

**Figure 2:**
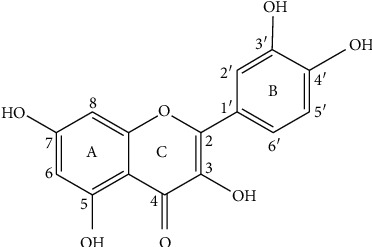
Quercetin structure.

Quercetin (3,3′,4′,5,7-pentahydroxyflavone) is one of the flavonoids which attracted more attention in recent years (Figure 2) [11]. This phenolic compound is a dietary flavonoid which existed in onions, apples, broccoli, berries, tomato, and lettuce, linked to the cell wall matrix [11–15]. Quercetin is a bioflavonoid that can protect tissues from injury induced by some drug toxicities [16].

Quercetin has poor solubility, so it seems to be difficult to absorb into the body [17, 18]. Several studies have been performed to modify the quercetin structure to increase its water solubility and bioavailability and thus enhance its pharmacological effects [19–23]. Studies have shown that coordination of quercetin with metal ions can increase the antioxidant activity and ultraoxygen anion elimination than quercetin itself [24–26]. The strong ability of quercetin to chelate with different metal ions such as Tb(III) [27], Mg(II) [28], Cu(II) [29], Fe(II) [3], Cr(III) [30], Co(II) [31], Sn(II) [32], Vo(IV) [33], Zn(II) [34], Mn(II) [35], Pb(II) [36], and Ni(II) [37] can increase the solubility and bioavailability of quercetin and promote new pharmacological activity [38].

Quercetin has two aromatic rings and an oxygenated heterocyclic ring containing a carbonyl group at 4-position and hydroxyl group at 3-carbon chain [39]. Functional hydroxyl groups in the flavonoids cause antioxidant activity by scavenging free radicals and chelating metal ions. The chelation of metals can prevent radical generation which damages target biomolecules. Naidu and Kinthada [40] showed that quercetin and quercetin-3-glycoside can react with thiosemicarbazide in methanol and produce thiosemicarbazone derivatives that can form stable complexes in reaction with some transition metals.

Interaction of quercetin with metal ions can change its antioxidant and biological activities due to the ability of this complex as a free radical scavenger [41, 42]. Studies show that the 3′,4′-ortho-dihydroxy substitution in the B ring is critical for copper ion chelation with quercetin to increase the antioxidant activity [43].

Copper is a bio-essential element for all organisms. It is used as a metal cofactor by some enzymes, including cytochrome c oxidase (Cox) and superoxide dismutase (SOD). In the body, copper is present in Cu^+^ and Cu^2+^ forms. It acts as an intermediary for electron transfer in redox reactions. Copper is a critical element for neuronal function and oxygen transport and a cofactor for many proteins [44–46] and acts as a cofactor in blood for angiogenesis [41].

Copper complexes are getting more attention due to their multiple bioactivities in living organism. Copper (II) complexes play significant role in enhancing the pharmacological profile of the antimicrobial activities of some bioactive compounds [42].

To date, the complexity of nanostructures has become interesting for fundamental and practical studies. Rational design of complex nanostructures can make new desired materials with special properties [47].

In this study, we first focused on the synthesis of Schiff base from the reaction between quercetin and ethanol amine and subsequently synthesized novel nanoscale Cu (II) complex as an excellent catalyst for alcohol oxidation. We investigated the potential catalytic activity of the synthesized catalyst in primary and secondary alcohol oxidation under solvent-free conditions. These results showed that the catalyst performs highly efficiently and due to its heterogeneous nature, it can be used several times in the chemical reactions. The experimental results also showed the synthesized nanoparticles have high antibacterial activity.

## 2. Materials and Methods

Quercetin, ethanolamine, and all solvents and reagents were purchased from Sigma-Aldrich. All chemicals were used without any further purification. The progress of the reactions and the purity of the products were monitored by TLC (thin layer chromatography). Fourier transform infrared (FT-IR) spectra were recorded with a Nicolet System 800 beam splitter in the range 400–4000 cm^−1^. NMR spectra were recorded on Bruker Avance 400 Ultrashield NMR spectrometers using tetramethylsilane as an internal standard. The powder X-ray diffraction pattern (XRD) of the final product was obtained with an X'Pert Pro-MPD diffractometer between 2*θ* = 2°–80°. Inductively coupled plasma (ICP) atomic emission spectroscopy was carried out by using OPTIMA 7300DV. FESEM analysis was carried out by MIRA TESCAN instrument to determine the morphology of the nanoparticles.

### 2.1. Schiff Base Synthesis

**Scheme 1:**
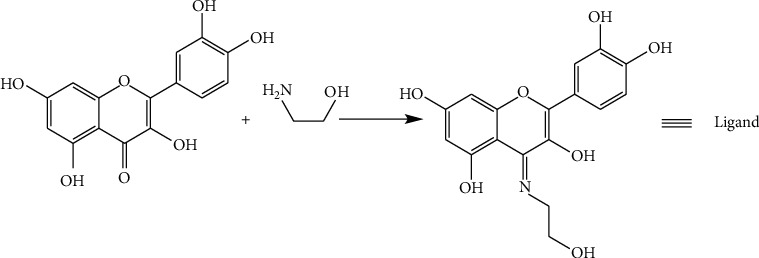
Synthesis of the Schiff base ligand.

The synthesis of Schiff base as a ligand is shown (Scheme 1). Quercetin (0.302 g, 1 mmol) was dissolved in ethanol (7 ml). Glacial acetic acid (57 µl) was added to this solution. Ethanol amine (60.2 *µ*l, 1 mmol) was added dropwise to the reaction flask after 30 minutes. The reaction mixture was refluxed at 60°C for 8 hours with stirring. The resulting dark red solution was concentrated and cooled, giving an orange crystalline precipitate after recrystallization from a hot solution of ethanol and dried in vacuo. The colour of the Schiff base was light orange.

### 2.2. Synthesis of Nanoscale Cu (II) Complex

**Scheme 2:**
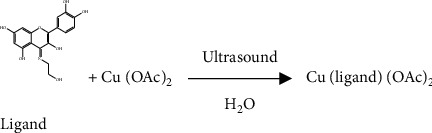
Synthesis of the novel complex.

The nanoscale Cu (II) complex was synthesized according to a one-pot strategy. First, the ligand (0.173 g, 0.5 mmol) was dissolved by adding NaOH 10% (1 ml) in deionized water (20 ml) under magnetic stirring for 10 minutes at room temperature to obtain an orange solution. Then, a solution of Cu(OAc)_2_ (0.091 g, 0.5 mmol) in deionized water (10 ml) was added dropwise to the mixture under ultrasonic irradiation at room temperature and was sonicated for 30 min. The resulting brown mixture was centrifuged and placed in a vacuum oven at 80°C for 6 hours, yielding dark orange precipitation (Scheme 2).

### 2.3. Catalytic Procedure for Alcohol Oxidation

The synthesized copper (II) complex was tested as a catalyst in benzyl alcohol oxidation to determine its catalytic activity. Several experiments were carried out to optimize temperature, solvent, and mol% of catalyst. The best result in the model reaction was chosen. In order to the investigation of solvent nature, the model experiment was carried out in different solvents and the best result was taken in solvent-free conditions. It seems that open catalytic sites in this condition are the reason of this observation. In the second step, the model reaction was carried out at different temperatures. The reaction yield at room temperature, 40°C and 50°C, was 98%, so the reaction was carried out at room temperature. Then, the catalyst and oxidant amount were investigated. The best yield was obtained in the presence of 1 mol% catalyst and 1 mmol of TBHP. For the investigation of oxidant type, the reaction was performed with H_2_O_2_, TBHP, and NaIO_4_. With the optimized reaction conditions, the best experiment as a model was chosen. 1 mol% of catalyst, 0.10 mL (1 mmol) of benzyl alcohol, and 0.096 mL (1 mmol) of tert-butyl hydroperoxide (TBHP) as an oxidant were mixed and stirred in solvent-free condition at room temperature for half an hour. After the determination of optimum condition, the ability of the catalyst for a series of alcohols was evaluated (Table 1).

The catalytic activity of the synthesized nanoparticles was compared with some other catalysis in the alcohol oxidation reaction (Table 2).

### 2.4. Antibacterial Activity

The synthesized complex was tested for in vitro antibacterial activity using the agar well diffusion method. *Staphylococcus aureus* (Gram‐positive) and *Escherichia coli* (Gram‐negative) were used as a model for this activity. The fresh bacteria were incubated on nutrient agar plates at 37°C for 24 hours. A nutrient agar for the plates was provided by dissolving agar powder (15.0 g L^−1^), tryptone (5.0 g L^−1^), yeast extract (2.5 g L^−1^), and glucose (1.0 g L^−1^) in deionized water. The pH of the mixture was adjusted to 7.0 ± 0.1. The diameter of the zone of inhibition was measured.

Gentamicin was used as a control substance. The antibacterial efficacy of the complex was tested against *Staphylococcus aureus* and *Escherichia coli* bacteria. The biocidal effect of the copper complex was investigated by the diffusion method. A bacterial suspension with a concentration of 5 × 10^5^ cfu/mL (200 mL) was spread on the agar plates. After the agar plates were incubated at 37°C for 48 hours, the synthesized complex (1 mg) was disposed directly into the holes (7 mm diameter) which were punched over the agar plates previously.

The evaluation of the antibacterial activity of the complex was carried out by the standard zone of inhibition test. The diameter of the zone of inhibition was measured in mm by the ruler.

## 3. Results and Discussion

The formation of a Schiff base between quercetin and ethanolamine was investigated by NMR spectroscopy. NMR data showed the formation of Schiff base from quercetin and ethanol amine.

**Figure 3:**
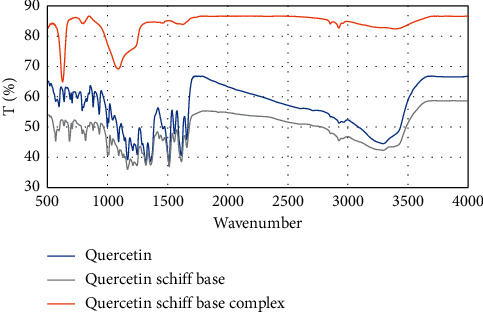
FT-IR spectrum of quercetin, quercetin Schiff base, and quercetin Schiff base complex.

The binding properties and the coordination sites were investigated by using IR spectroscopy. The main peaks of quercetin, quercetin Schiff base, and quercetin Schiff base complex are shown in Table 3. Important information was obtained by comparing the quercetin with the Schiff base and the complex. It was observed that C=O stretching mode of free quercetin occurred at 1707 cm^−1^, which was shifted to 1695 cm^−1^ in the Schiff base, and it was shifted to 1622 cm^−1^ by the formation of the complex. A sharp peak at 1612 cm^−1^ shows the formation of the imine band in Schiff base. The sharp stretching vibration at 625 cm^−1^ shows Cu-O chelation indicating the copper (II) is chelated to quercetin Schiff base ligand and formed the metal complex while this stretching band was absent in the FT-IR spectrum of the ligand. The broad medium intensity band at the frequency range of 3200–3450 cm^−1^ may be assigned to the -OH group. In the quercetin Schiff base copper complex, a broad -OH group was observed as broad vibrations around 3300 cm^−1^ (Figure 3).

### 3.1. Schiff Base Complex

^1^H NMR spectra of quercetin Schiff base was obtained by using DMSO as a solvent and the main data are reported as follows: *δ* 3.97 (1H, s), 4.64 (2H, t), 5.90 (2H, m), 6.43 (1H, d), 7.00 (1H, dd), 7.52 (2H, dd), 9.58 (2H, s), 10.05 (3H, s), and 10.71 (1H, s) (Figure S1). The ^13^C NMR spectral data of the ligand were recorded in DMSO solution. In the ^13^C NMR spectrum, characteristic peaks indicate the formation of Cu(II) complex. The main data are reported as follows: *δ* 56.48 (C attached to the OH group of ethanolamine), 65.78 (C attached to the nitrogen in ethanolamine), 94.02 (C_8_), 99.05 (C_6_), 102.89 (C between C_4_ and C_5_), 115.43 (C_2′_), 116.12 (C_5′_), 120.35 (C_6′_), 122.42 (C_1′_), 128.78 (C_3_), 145.65 (C_3′_), 146.93 (C_4′_), 148.33 (C_2_), 156.73 (C between C_8_ and O in C ring), 162.85 (C_7_), 164.28 (C_5_), and 166.09 (C_4_) (Figure S2).

**Figure 4:**
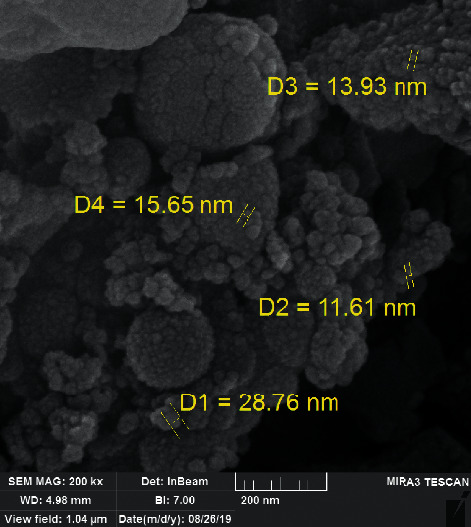
FESEM image of the new nanoscale Cu (II) Schiff base complex.

The synthesized nanoscale copper (II) complex was confirmed by the EDX spectrum and FESEM image measurement (Figure 4), which confirmed the highest formation percent of Cu (II) complex. FESEM image for nanoscale copper (II) complex showed nanoparticles with an average diameter of 17 nm.

**Figure 5:**
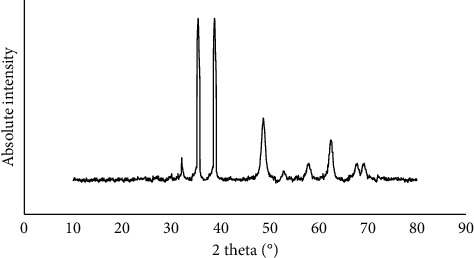
XRD pattern of the new nanoscale Cu (II) Schiff base complex.

XRD of synthesized nanoscale copper (II) complex gives characteristic peaks at 2*θ* = 32.12°, 35.37°, 38.77°, 48.67°, 53.37°, 57.92°, 62.52°, 67.62°, and 69.12^°,^ indicating the formation of Cu(II) complex (Figure 5) [55].

**Figure 6:**
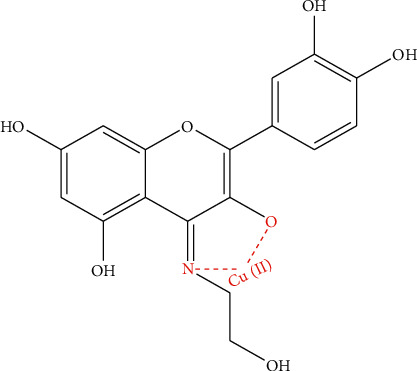
Possible structure of the complex.

As the ratio of metal ion and ligand is 1 : 1 and the 3-hydroxy group has a more acidic proton, the 3-OH and N positions are the best site to be involved in the complexation process [29, 56]. The OH group from the alcohol is not coordinated, possibly due to the distance between OH group and the center of reaction. Similar study confirms this structure [57] (Figure 6).

**Scheme 3:**
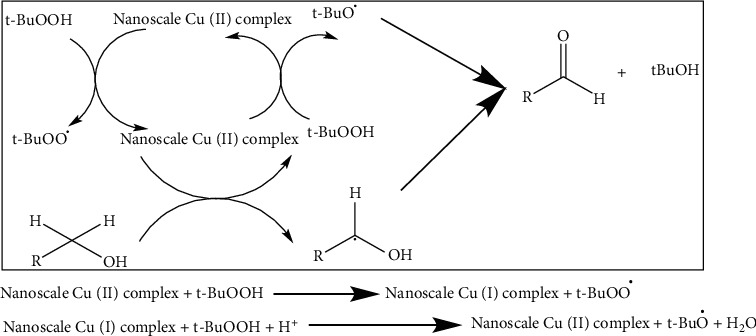
Proposed mechanism for the alcohol oxidation by synthesized catalyst.

The oxidation of alcohols mechanism may involve the generation of tBuOO^•^ and t-BuO^•^ radicals by the metal assisted, which behave as hydrogen atom abstractors from the alcohols. The ligand can assist proton transfer steps involved in the fundamental steps of the alcohol oxidation reaction. The mechanism is summarized in Scheme 3.

After optimizing reaction conditions, the effect of a catalyst in a series of primary and secondary alcohols including aromatic ring and electron donating and withdrawing groups was evaluated. The results are summarized in the table. In general, all substrates showed an excellent yield in alcohol oxidation reactions. Electron donating and electron withdrawing groups change the reaction yield slightly.

The recyclability of the catalyst was tested in the alcohol oxidation reaction. The complex was recovered from the reaction after three times for the next reaction run by centrifugation, washing, and drying in the oven. This catalyst was reused three times without any significant change of catalytic activity.

The synthesized complex was tested for the in vitro antibacterial activity against *E. coli* (Gram-negative) and *Staphylococcus aureus* (Gram-positive) bacteria at concentration of 100 mM. The complex was active against both of these bacteria. The results of the antibacterial activity are reported as inhibition zone diameter (mm).

The antibacterial activity of the complex can be attributed to the involvement of metal ions as a candidate for bacterial growth inhibition, which could be explained by chelation theory [58].

Gram-positive bacteria have lipopolysaccharides cell wall. This wall prevents the accumulation of the complex in the cell membrane. Therefore, Gram-positive bacteria are more effective and more sensitive compared to Gram-negative bacteria.

**Figure 7:**
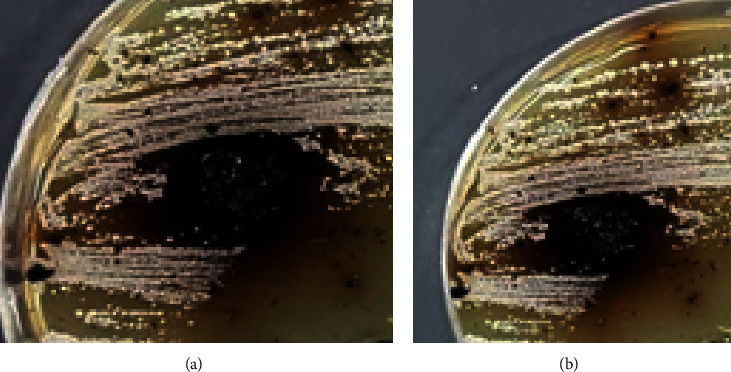
Inhibition areas for the synthesized nanoscale Cu (II) complex. (a) *E. coli* and (b) *Staphylococcus aureus* by agar-well diffusion method (concentration used: 100 mM) in open condition.

Transition metal complexes have an important place in biochemistry [38, 59]. The antibacterial activity of the synthesized complex was evaluated and compared with standard (gentamicin) (Figure 7). The diameter of the zone showing complete inhibition is listed (Table 4).

This study clearly showed that synthesized complex has reasonable antibacterial activity against both Gram-negative and positive organisms. Enhanced lipophilic properties of metal ion sites caused the high antibacterial activity of the synthesized complex. The enhanced lipophilicity led to cell death by easy translocation of nanoscale Cu (II) complex.

## 4. Conclusions

In this study, formation of Schiff base between quercetin and ethanolamine is investigated. The reaction was carried out in ethanolic solution under reflux condition. Quercetin is a flavonoid with potent antioxidant activity and broad clinical effect. The biological activities of quercetin increase when it is coordinated with metal ions. This is due to the fact that after forming the complex, solubility and bioavailability of quercetin in the body are increased. In the second step, a novel nanoscale Cu (II) complex was synthesized, and its catalyst effect in oxidation reactions of alcohols was examined. Copper is a bioactive metal that plays several roles in biological processes, such as catalyzing a large number of biochemical reactions and having a key role in electron transport in mitochondria. Copper (II) complexes show a wide variety of biological activities. They could be used as antimicrobial, anti-inflammatory, antitumor, and antiviral agents. Therefore, Cu(II) complexes are synthesized as a potential drug. The spectroscopic data showed the 3-OH group and imine groups are coordination sites with the metal ion.

The alcohol oxidation reaction was performed in green condition that is in accordance with environmentally friendly protocols. The catalyst is highly stable and can be reused several times without the loss of catalytic activity in the alcohol oxidation reaction.

Furthermore, the synthesized complex indicated promising antibacterial activity against *E. coli* (Gram-negative) and *Staphylococcus aureus* (Gram-positive) bacteria. *Staphylococcus aureus* has higher antibacterial activity than *E. coli* due to the differences between cell structure, metabolism, and physiology of Gram-positive and Gram-negative bacteria. These factors are influential on the sensitivity of the nanoscaled copper complex on the antibacterial activity.

## Figures and Tables

**Table 1:** Oxidation reactions of various alcohols catalysed by the synthesized catalyst^a^.

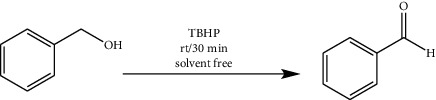				
Entry	Substrate	Product	Time (min.)	Yield^b^ (conv.) %
1	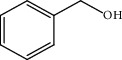	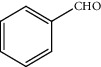	30	98
2	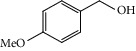	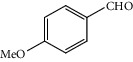	30	95
3	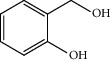	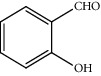	30	91
4	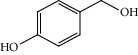	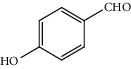	30	93
5	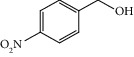	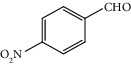	30	90
6	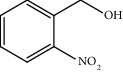	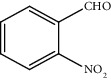	30	88
7	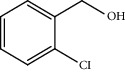	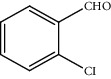	30	85
8	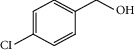	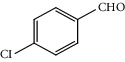	30	87
9	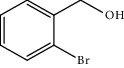	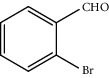	30	86
10	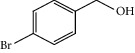	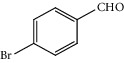	30	84
11	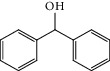	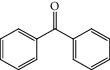	30	80
12	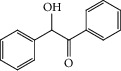	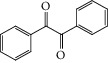	30	82

^a^Reaction conditions: alcohols (1 mmol), TBHP (1 mmol), and synthesized complex (1 mol%) at room temperature and the absence of any solvent. ^b^Isolated yield.

**Table 2:** Comparison of catalytic activity in alcohol oxidation reaction with previously reported catalysts.

Entry	Catalyst	Catalyst amount	Condition	Time (h)	Yield (%)	Ref.
1	Cu-MOF-2	17 mol%	CH_3_CN/O_2_/75°C	16	99	[43]
2	UiO-66-Sal-CuCl_2_	4 mol%	CH_3_CN/O_2_/60°C	24	99	[47]
3	[Co_3_L(PTA)_2.5_(OAc)]^b^	2 mol%	CH_3_CN/TBHP/60°C	24	88	[48]
4	[{Cu(L1)- (DMF)}·DMF·H_2_O]_n_^a^	0.2 mol%	MW/TBHP/100°C	0.5	81.1	[48]
5	Cu_3_(BTC)_2_	5 mol%	CH_3_CN/O_2_/70°C	9	94	[49]
6	Au@Cu(II)-MOF	3 mol%	Toluene/air/110°C	15	98	[50]
7	Cu-MOF-74	2.5 mol%	CH_3_CN/O_2_/70°C	12	89	[51]
8	SPS–Cu (II)@Cu_3_(BTC)_2_	2 mol%	CH_3_CN/O_2_/75°C	8	99	[52]
9	Pd@Cu(II)- MOF	5 mol%	Xylene/air/130°C	25	95	[53]
10	MIL-53(Fe)-graphene	15 mol%	CCl_4_/visible light	9	80	[54]
11	Synthesized nanoscale Cu (II) complex	1 mol%	SF/TBHP/rt	0.5	98	This work

^a^PTA, p-phthalic acid; ^b^L_1_, 5-{(pyridin-4-ylmethyl)amino} isophthalic acid.

**Table 3:** Comparison of main peaks in quercetin, quercetin Schiff base, and quercetin Schiff base complex.

Compound	V (C=O)	V (C=C)	V (O-H)	V (C-O-C)	V (Cu-O)
Quercetin	1707	1554	3296	1277	Absent
Quercetin Schiff base	1695	1558	3308	1282	Absent
Quercetin Schiff base complex	1622	1578	3415	1079	625

**Table 4:** Diameter of the halos in two types of testing bacteria.

Bacteria	*E. coli* (Gram-negative) (mm)	*Staphylococcus aureus* (Gram‐positive) (mm)
Inhibition of bacterial growth by the nanoscale Cu (II) complex	16	18

## Data Availability

The data used to support the findings of this study are available from the corresponding author upon request.

## Supplementary Materials

Supplementary Materials Figure S1: ^1^H NMR Schiff base as a ligand. Figure S2: ^13^C NMR Schiff base as a ligand.

## References

[1] Dueñas M., González-Manzano S., González-Paramás A., Santos-Buelga C. (2010). Antioxidant evaluation of O-methylated metabolites of catechin, epicatechin and quercetin. *Journal of Pharmaceutical and Biomedical Analysis*.

[2] Rattanachaikunsopon P., Phumkhachorn P. (2010). Contents and antibacterial activity of flavonoids extracted from leaves of Psidium guajava. *Journal of Medicinal Plant Research*.

[3] Raza A., Xu X., Xia L., Xia C., Tang J., Ouyang Z. (2016). Quercetin-Iron complex: synthesis, characterization, antioxidant, DNA binding, DNA cleavage, and antibacterial activity studies. *Journal of Fluorescence*.

[4] Rauf A., Imran M., Khan I. A. (2018). Anticancer potential of quercetin: a comprehensive review. *Phytotherapy Research*.

[5] Yang Z., Liu Y., Liao J. (2015). Quercetin induces endoplasmic reticulum stress to enhance cDDP cytotoxicity in ovarian cancer: involvement of STAT3 signaling. *The FEBS Journal*.

[6] Nguyen T. T., Tran E., Nguyen T. H., Do P. T., Huynh T. H., Huynh H. (2004). The role of activated MEK-ERK pathway in quercetin-induced growth inhibition and apoptosis in A549 lung cancer cells. *Carcinogenesis*.

[7] Jia Q., Cao H., Shen D (2019). Quercetin protects against atherosclerosis by regulating the expression of PCSK9, CD36, PPAR*γ*, LXR*α* and ABCA1. *International Journal of Molecular Medicine*.

[8] Costa L. G., Garrick J. M., Roquè P. J., Pellacani C. (2016). Mechanisms of neuroprotection by quercetin: counteracting oxidative stress and more. *Oxidative Medicinal Cell Longevity*.

[9] Wang T.-y., Li Q., Bi K.-S. (2018). “Bioactive flavonoids in medicinal plants: structure, activity and biological fate. *Asian Journal of Pharmaceutical Sciences*.

[10] Kumar S., Pandey A. K. (2013). Chemistry and biological activities of flavonoids: an overview. *Scientific World Journal*.

[11] Wang W., Sun C., Mao L. (2016). The biological activities, chemical stability, metabolism and delivery systems of quercetin: a review. *Trends in Food Science and Technology*.

[12] Slimestad R., Fossen T., Verheul M. J. (2008). The flavonoids of tomatoes. *Journal of Agricultural and Food Chemistry*.

[13] Materska M., Olszówka K., Chilczuk B. (2019). Polyphenolic profiles in lettuce (*Lactuca sativa* L.) after CaCl_2_ treatment and cold storage. *European Food Research and Technology*.

[14] Lakhanpal P., Rai D. K. (2007). Quercetin: a versatile flavonoid. *Internet J Med Updat - EJOURNAL*.

[15] Huynh N. T., Smagghe G., Gonzales G. B., Van Camp J., Raes K. (2018). Bioconversion of kaempferol and quercetin glucosides from plant sources using Rhizopus spp. *Fermentation*.

[16] Anand David A. V., Arulmoli R., Parasuraman S. (2016). Overviews of biological importance of quercetin: a bioactive flavonoid. *Pharmacognosy Reviews*.

[17] Xu D., Hu M. J., Wang Y. Q., Cui Y. L. (2019). Antioxidant activities of quercetin and its complexes for medicinal application. *Molecules*.

[18] Kasikci M. B., Bagdatlioglu N. (2016). Bioavailability of quercetin. *Current Research in Nutrition and Food Science*.

[19] Chen X., Yin O. Q. P., Zuo Z., Chow M. S. S. (2005). Pharmacokinetics and modeling of quercetin and metabolites. *Pharmaceutical Research*.

[20] Massi A., Bortolini O., Ragno D. (2017). Research progress in the modification of quercetin leading to anticancer agents. *Molecules*.

[21] Park K. H., Choi J. M., Cho E. (2017). Enhancement of solubility and bioavailability of quercetin by inclusion complexation with the cavity of mono-6-deoxy-6-aminoethylamino-*β*-cyclodextrin. *Bulletin of the Korean Chemical Society*.

[22] Dian L., Yu E., Chen X. (2014). Enhancing oral bioavailability of quercetin using novel soluplus polymeric micelles. *Nanoscale Research Letter*.

[23] Amanzadeh E., Esmaeili A., Ren A., Kazemipour N., Pahlevanneshan Z., Beheshti S. (2019). Quercetin conjugated with superparamagnetic iron oxide nanoparticles improves learning and memory better than free quercetin via interacting with proteins involved in LTP. *Scientific Reports*.

[24] Ravichandran R., Rajendran M., Devapiriam D. (2014). Antioxidant study of quercetin and their metal complex and determination of stability constant by spectrophotometry method. *Food Chemistry*.

[25] De Souza R. F. V., Sussuchi E. M., De Giovani W. F. (2003). Synthesis, electrochemical, spectral, and antioxidant properties of complexes of flavonoids with metal ions. *Synthesis and Reactivity in Inorganic and Metal-Organic Chemistry*.

[26] Oliveira C. G., Maia P. I. D. S., Miyata M. (2014). Cobalt(III) complexes with thiosemicarbazones as potential anti-Mycobacterium tuberculosis agents. *Journal of the Brazilian Chemical Society*.

[27] Jen D., Mokhtarzadeh A., Ghareghoran S. M., Dehghan G. (2014). Synthesis, characterization and antioxidant property of Quercetin-Tb(III) complex. *Advanced Pharmaceutical Bulletin*.

[28] Ghosh N., Chakraborty T., Mallick S. (2015). Synthesis, characterization and study of antioxidant activity of quercetin-magnesium complex. *Spectrochimica Acta Part A: Molecular and Biomolecular Spectroscopy*.

[29] Bukhari S. B., Memon S., Mahroof-Tahir M., Bhanger M. I. (2009). Synthesis, characterization and antioxidant activity copper-quercetin complex. *Spectrochimica Acta Part A: Molecular and Biomolecular Spectroscopy*.

[30] Chen W., Sun S., cao W., Liang Y., Song J. (2009). Antioxidant property of quercetin-Cr(III) complex: the role of Cr(III) ion. *Journal of Molecular Structure*.

[31] Trifunschi S., Ardelean D. (2016). Synthesis, characterization and antioxidant activity of Co(II) and Cd(II) complexes with quercetin. *Revista de Chimie*.

[32] Dehghan G., Khoshkam Z. (2012). Tin(II)-quercetin complex: synthesis, spectral characterisation and antioxidant activity. *Food Chemistry*.

[33] Ferrer E. G., Salinas M. V., Correa M. J. (2006). Synthesis, characterization, antitumoral and osteogenic activities of quercetin vanadyl(IV) complexes. *JBIC Journal of Biological Inorganic Chemistry*.

[34] Tan J., Wang B., Zhu L. (2009). DNA binding, cytotoxicity, apoptotic inducing activity, and molecular modeling study of quercetin zinc(II) complex. *Bioorganic and Medicinal Chemistry*.

[35] Jun T., Bochu W., Liancai Z. (2007). Hydrolytic cleavage of DNA by quercetin manganese(II) complexes. *Colloids and Surfaces B: Biointerfaces*.

[36] Ravichandran R., Rajendran M., Devapiriam D. (2014). Structural characterization and physicochemical properties of quercetin-Pb complex. *Journal of Coordination Chemistry*.

[37] Alper P., Erkisa M., Genckal H. M., Sahin S., Ulukaya E., Ari F. (2019). Synthesis, characterization, anticancer and antioxidant activity of new nickel(II) and copper(II) flavonoid complexes. *Journal of Molecular Structure*.

[38] Zhang L.-J., Zhang J.-A., Zou X.-Z. (2015). Studies on antimicrobial effects of four ligands and their transition metal complexes with 8-mercaptoquinoline and pyridine terminal groups. *Bioorganic and Medicinal Chemistry Letters*.

[39] Shaker A. N. A.-J., Al-Diwan M. A., Sami Mathdi A. (2018). Synthesis, characterization and antioxidant activity of novel quercetin derivative. *Life Science Architecture*.

[40] Naidu S., Kinthada P. M. (2012). Structure and biological activities of novel phytochemicals Cu(Ii)-Quercetin thiosemicarbazone and its derivatives: potential anti-cancer drugs. *International Journal of Pharma and Bio Science Subrahmanyam Naidu P V Prakash MMS Kinthada*.

[41] Tripathy D. R., Singha Roy A., Dasgupta S. (2011). Complex formation of rutin and quercetin with copper alters the mode of inhibition of Ribonuclease A. *FEBS Letters*.

[42] Liu H., Yang W., Zhou W., Xu Y., Xie J., Li M. (2013). Crystal structures and antimicrobial activities of copper(II) complexes of fluorine-containing thioureido ligands. *Inorganica Chimica Acta*.

[43] Qi Y., Luan Y., Yu J., Peng X., Wang G. (2015). Nanoscaled copper metal-organic framework (MOF) based on carboxylate ligands as an efficient heterogeneous catalyst for aerobic epoxidation of olefins and oxidation of benzylic and allylic alcohols. *Chemistry-A European Journal*.

[44] Siotto M., Squitti R. (2018). Copper imbalance in Alzheimer’s disease: overview of the exchangeable copper component in plasma and the intriguing role albumin plays. *Coordination Chemistry Reviews*.

[45] Manto M. (2014). Abnormal copper homeostasis: mechanisms and roles in neurodegeneration. *Toxics*.

[46] Baker Z. N., Cobine P. A., Leary S. C. (2017). The mitochondrion: a central architect of copper homeostasis. *Metallomics*.

[47] Hou J., Luan Y., Tang J., Wensley A. M., Yang M., Lu Y. (2015). Synthesis of UiO-66-NH2 derived heterogeneous copper (II) catalyst and study of its application in the selective aerobic oxidation of alcohols. *Journal of Molecular Catalysis A: Chemical*.

[48] Karmakar A., Martins L. M. D. R. S., Hazra S., Guedes da Silva M. F. C., Pombeiro A. J. L. (2016). Metal-organic frameworks with pyridyl-based isophthalic acid and their catalytic applications in microwave assisted peroxidative oxidation of alcohols and henry reaction. *Crystal Growth & Design*.

[49] Kim B. R., Oh J. S., Kim J., Lee C. Y. (2016). Robust aerobic alcohol oxidation catalyst derived from metal-organic frameworks. *Catalysis Letters*.

[50] Wang J.-S., Jin F.-Z., Ma H.-C. (2016). Au@Cu(II)-MOF: highly efficient bifunctional heterogeneous catalyst for successive oxidation-condensation reactions. *Inorganic Chemistry*.

[51] Kim B. R., Oh J. S., Kim J., Lee C. Y. (2015). Aerobic oxidation of alcohols over copper-containing metal-organic frameworks. *Bulletin of the Korean Chemical Society*.

[52] Zhang X., Dong W., Luan Y. (2015). Highly efficient sulfonated-polystyrene-Cu(II)@Cu3(BTC)2 core-shell microsphere catalysts for base-free aerobic oxidation of alcohols. *Journal of Materials Chemistry A*.

[53] Chen G.-J., Wang J.-S., Jin F.-Z. (2016). Pd@Cu(II)-MOF-Catalyzed aerobic oxidation of benzylic alcohols in air with high conversion and selectivity. *Inorganic Chemistry*.

[54] Yang Z., Xu X., Liang X. (2016). MIL-53(Fe)-graphene nanocomposites: efficient visible-light photocatalysts for the selective oxidation of alcohols. *Applied Catalysis B: Environmental*.

[55] Raul P. K., Senapati S., Sahoo A. K. (2014). CuO nanorods: a potential and efficient adsorbent in water purification. *RSC Advances*.

[56] Li T.-R., Yang Z.-Y., Wang B.-D. (2007). Synthesis, characterization and antioxidant activity of naringenin Schiff base and its Cu(II), Ni(II), Zn(II) complexes. *Chemical & Pharmaceutical Bulletin*.

[57] Stetsiuk O., Plyuta N., Avarvari N. (2020). Mn(III) chain coordination polymers assembled by salicylidene-2-ethanolamine Schiff base ligands: synthesis, crystal structures, and HFEPR study. *Crystal Growth and Design*.

[58] Srivastava R. S. (1981). Pseudotetrahedral Co(II), Ni(II) and Cu(II) complexes of N1-(O-chlorophenyl)-2-(2′,4′-dihydroxyphenyl)-2-benzylazomethine their fungicidal and herbicidal activity. *Inorganica Chimica Acta*.

[59] Legin A. A., Jakupec M. A., Bokach N. A., Tyan M. R., Kukushkin V. Y., Keppler B. K. (2014). Guanidine platinum(II) complexes: synthesis, in vitro antitumor activity, and DNA interactions. *Journal of Inorganic Biochemistry*.

