# Supplementary material for: Quercetin as a Precursor for the Synthesis of Novel Nanoscale Cu (II) Complex as a Catalyst for Alcohol Oxidation with High Antibacterial Activity
Source: Bioinorg Chem Appl. 2021 Mar 3;2021:8818452. doi: 10.1155/2021/8818452 (PMC7952193; doi:10.1155/2021/8818452)
Supplement: Supplementary Materials — Figure S1: 1H NMR Schiff base as a ligand. Figure S2: 13C NMR Schiff base as a ligand. [file 8818452.f1.docx]

**Bioinorganic Chemistry and Applications**

Supporting Information

**Quercetin as a precursor for the synthesis of novel nanoscale Cu (II) complex as a catalyst for alcohol oxidation with high antibacterial activity**

Zahra Moodi,GhodsiehBagherzade^*^,Janny Peters

**SupportingInformation**

**Table of Contents**

| **S. No.** | **Particulars** | **Pages** |
| --- | --- | --- |
| 1 | ^1^H NMR Spectrum of the synthesized  Schiff base | 3 |
| 2 | ^13^C NMR Spectrum of the synthesized  Schiff base | 4 |

**^1^H NMR Spectrum of the synthesized Schiff base**


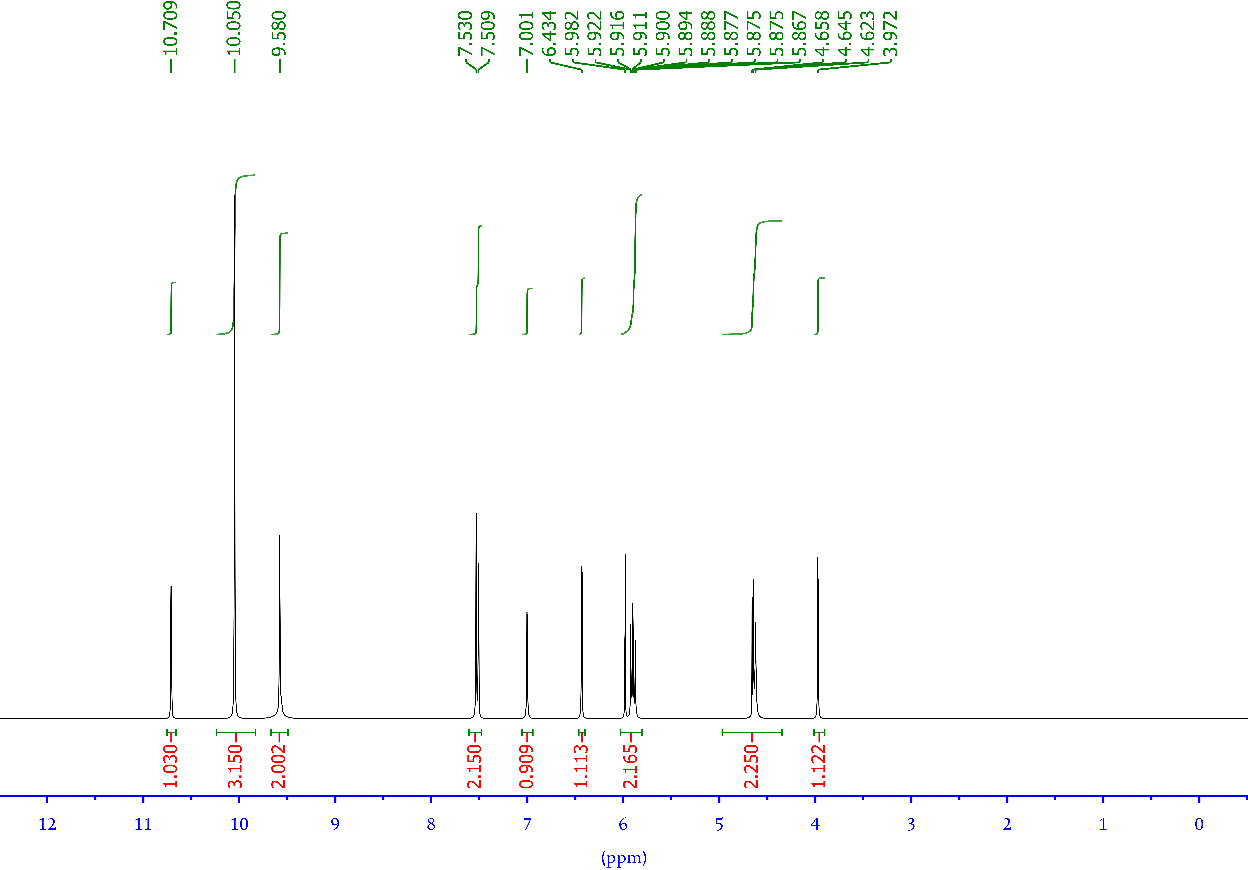


**FigureS1:** ^1^H NMR Schiff base as a ligand

**^13^C NMR Spectrum of the synthesized Schiff base**


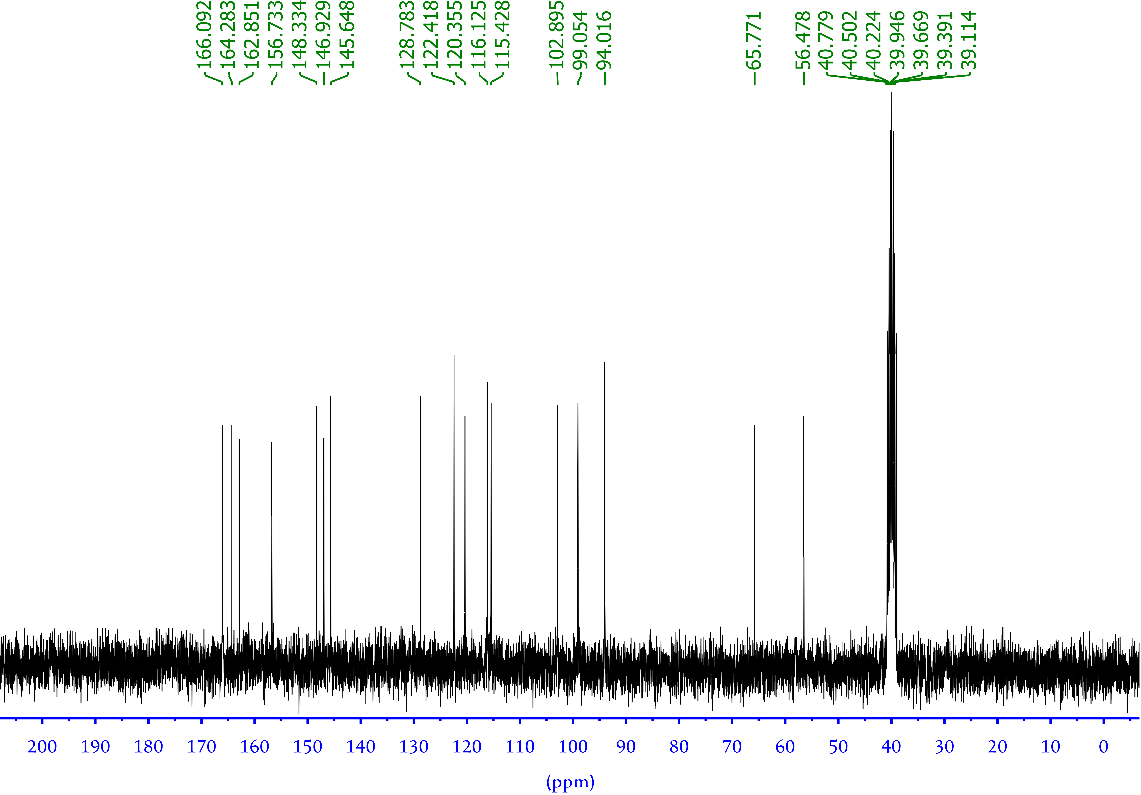


**FigureS2:**^13^C NMR Schiff base as a ligand
